# Identification of late blight resistance QTLs *in an interspecific RIL population of tomato *via genotyping-by-sequencing

**DOI:** 10.1007/s11032-025-01560-6

**Published:** 2025-04-08

**Authors:** Mengyuan Jia, Hudson Ashrafi, Majid R. Foolad

**Affiliations:** 1 Department of Plant Science and the Intercollege Graduate Degree Program in Plant Biology, The Pennsylvania State Univ, University Park, PA 16802 USA; 2 Department of Horticultural Science, North Carolina State University, Raleigh, NC 27695 USA

**Keywords:** Disease resistance, Genotyping-by-sequencing, *Phytophthora infestans*, QTL mapping, Recombinant inbred line, SNP, *Solanum lycopersicum*, *Solanum pimpinellifolium*

## Abstract

Late blight (LB), caused by *Phytophthora infestans*, is a destructive disease of the cultivated tomato, *Solanum lycopersicum*. Environmental concerns and pathogen resistance have propelled research towards developing host resistance. The current LB-resistant cultivars of tomato exhibit susceptibility under severe disease pressure, necessitating the identification, characterization, and incorporation of additional resistance genes into new tomato cultivars. Recently, we identified *Solanum pimpinellifolium* accession PI 270443 with strong resistance to LB and developed a RIL population from its cross with an LB-susceptible tomato breeding line. In the present study, we constructed a high-density genetic map of the RIL population, using 8,470 SNP markers set into 1,195 genomic bins, with a total genetic distance of 1232 cM and an average bin size of 1 cM. We identified 2 major adjoining LB-resistance QTLs on chromosome 10 and a few minor QTLs on chromosomes 1 and 12 of PI 270443. While one of the QTLs on chromosome 10 colocalized with the known LB-resistance gene *Ph- 2* and a LB-resistance QTL previously identified in an F_2_ population of the same cross, the present study allowed marker saturation of the region, fine mapping of the QTL, and identification of candidate resistance genes in the region. One of the 2 major QTLs on chromosome 10 and the 3 QTLs on chromosomes 1 and 12 were not previously reported in *S. pimpinellifolium* for LB resistance. These results will expedite transferring of LB resistance from PI 270443 into the tomato cultigen via MAS and discovering the underpinning LB-resistance genes in PI 270443.

**Supplementary Information:**

The online version contains supplementary material available at 10.1007/s11032-025-01560-6.

## Introduction

The cultivated tomato, *Solanum lycopersicum* L., is ranked the world’s most valuable vegetable crop, with a net production value of nearly $106 billion in 2022 (FAOSTAT 2025). However, tomato production is negatively affected by various pathogens, including bacteria, fungi, oomycetes, viruses, and nematodes.(Jones et al. 2014). One of the most destructive diseases of the cultivated tomato (and potato, *Solanum tuberosum* L.) worldwide is late blight (LB), caused by the oomycete *Phytophthora infestans* (Mont.) de Bary (Fry 2008). Under proper conditions, this pathogen can infect all above-ground parts of unprotected susceptible tomato or potato crops, and destroy an entire field within 7–10 days (Nowicki et al. 2012). Management of LB disease in both tomato and potato fields has primarily included cultural practices and heavy use of preventative fungicides (Fry 2008). The rapid spread of *P. infestans* is mainly due to its effective asexual reproduction, though it can also reproduce sexually when both mating types (A1 and A2) are present (Smoot et al. 1958; Galindo and Gallegly 1960). Improper fungicide applications and sexual reproduction can lead to new, fungicide-resistant genotypes of *P. infestans* (Foolad et al. 2014b). For example, in 2018, a new mefenoxam-resistant *P. infestans* clonal lineage (US- 25, A2 mating type) emerged in New York tomato fields (USAblight.org 2019). The occurrence of new pathogen genotypes with fungicide resistance have driven research into developing host resistance to combat LB disease in both tomato and potato crops.

Host resistance of plants can recognize and act toward late blight disease through two main pathways, either by Pattern Recognition Receptors (PRRs) detecting pathogen-associated molecular patterns (PAMPs) to trigger PAMP-Triggered Immunity (PTI), or through Resistance (R) proteins recognizing pathogen effectors inside plant cells, activating the Effector-Triggered Immunity (ETI). These two pathways can work together and activate downstream regulatory genes (e.g., transcription factors) and hormone signaling pathways, which collectively activate general defense mechanisms, reactive oxygen species (ROS) signaling and hypersensitive response (HR) to enhance resistance and limit pathogen spread in plants (Li et al. 2024). In both potato and tomato, many R proteins have been discovered to help plants detect pathogen-derived molecules called avirulence (Avr) proteins that are specific to *P. infestans*, and trigger hypersensitive response (Bhatia et al. 2024). In potato, more than 50 resistance (*R*) genes conferring LB resistance have been identified and mapped in 21 related wild species, of which more than 25 *R* genes have been cloned and characterized. Most of the cloned *R* genes for LB resistance in potato encode for nucleotide-binding leucine-rich repeat (NB-LRR) proteins (Rodewald and Trognitz 2013; Paluchowska et al. 2022). Stacking of multiple *R* genes has been an effective approach for developing potatoes with strong LB resistance (Jo et al. 2014; Ghislain et al. 2019).

Since the 1950 s, researches on tomato resistance to LB have identified three major resistance genes: *Ph- 1*, *Ph- 2*, and *Ph- 3* (Foolad et al. 2008). Briefly, *Ph- 1*, located on chromosome 7 in *S. pimpinellifolium* accessions West Virginia 19 and 731, confers resistance only to *P. infestans* race T0 (Gallegly and Marvel 1955; Peirce 1971) but not to the currently predominant T1 race (Cohen 2002; Elsayed et al. 2011). *Ph- 2*, originally identified in *S. pimpinellifolium* accession West Virginia 700 (Gallegly 1960), was fine-mapped to a 141-kb region on the distal end of the long arm of chromosome 10, and most recently has been confirmed (through sequence analysis and post-inoculation expression studies) to be the gene Solyc10 g085460 (RGA1) encoding a CC-NBS-LRR disease resistance protein (Moreau et al. 1998; Zhi et al. 2021; Pan et al. 2024). Tomato cultivars containing only *Ph- 2* exhibit partial resistance to various *P. infestans* isolates, including US- 13, US- 14 and US- 23 (Black et al. 1996; Moreau et al. 1998; Foolad et al. 2014b). *Ph- 3*, the most effective LB-resistance gene, is located on chromosome 9 in *S. pimpinellifolium* accession L3708 and encodes a CC-NBS-LRR protein (Black et al. 1996; Chunwongse et al. 2002; Zhang et al. 2014); it provides good resistance against several *P. infestans* clonal lineages, including US- 13, US- 14 and US- 23. However, severe LB symptoms have been observed in cultivars containing only *Ph- 3* in many field trials globally (Chunwongse et al. 2002) (MR Foolad, *unpublished*; RG Gardner, *personal communication*). Tomato genotypes with *Ph- 2* + *Ph- 3* combined exhibit the strongest resistance to LB (Foolad et al. 2014b; McGrath 2015), though still exhibit concerning LB symptoms under disease-favorable conditions (RG Gardner; *personal communication*). To combat more aggressive *P. infestans* genotypes, it is important to identify and incorporate additional LB-resistance genes/QTLs (quantitative trait loci) into new tomato breeding lines and hybrid cultivars.

Many QTL studies have also been conducted to identify new genomic regions and genes associated with LB resistance in wild tomato species *S. pimpinellifolium*, including PI 224710, PI 270441, PI 270442, PI 270443, PI 163245 (later classified as *S. lycopersicum*) by our research group (Merk et al. 2012; Ohlson et al. 2018; Sullenberger et al. 2022; Gao and Foolad 2024; Sullenberger and Foolad 2024). LB resistance QTLs have also been discovered by other research groups in accessions of *S. pimpinellifolium* (Chen et al. 2014) and *S. habrochaites* (Lough 2003; Brouwer et al. 2004; Brouwer and St. Clair 2004; Li et al. 2011; Chen et al. 2008).

To identify additional genetic sources of LB resistance in tomato, 67 *S. pimpinellifolium* accessions were screened and accession PI 270443 was identified with strong resistance, comparable to the resistant line NC 03220 that contains *Ph- 2* and *Ph- 3* combined (Foolad et al. 2014b, 2015; Jia and Foolad 2019). To investigate the genetic basis of LB resistance in PI 270443, the wild accession was crossed with LB-susceptible NC EBR- 2 and produced an F_2_ population. Preliminary QTL analysis using 54 F_2_ individuals and 153 DNA markers identified two resistance QTL regions: *Ph- 5.1* on chromosome 1, and *Ph- 5.2* on chromosome 10 (Merk et al. 2012). Both QTL regions encompassed very large genetic and physical map distances, including many genes, presenting obstacles in fine mapping and gene cloning.

To further investigate the nature of LB resistance in PI 270443, we recently developed an advanced recombinant inbred line (RIL) population from the above-mentioned cross (Jia and Foolad 2019) Subsequently, using different generations (F_9_ and F_10_) of this RIL population, we conducted parent:offspring regression/correlation analyses to estimate heritability (*h*^*2*^) of LB resistance; our analyses showed a high *h*^*2*^ (0.76) for resistance in PI 270443 (Jia and Foolad 2019). The present study was aimed to develop a high-density genetic linkage map of the RIL population, identify and delineate QTLs for LB resistance in PI 270443, and identify candidate resistance genes residing in the QTL regions.

Next-generation sequencing (NGS) techniques have made genetic mapping more cost-effective, especially for detecting single nucleotide polymorphism (SNP) using whole-genome resequencing, SNP arrays, or genotyping-by-sequencing (GBS) approaches. In the current study we chose the GBS approach to identify SNP markers for genetic mapping in the RIL population. This method uses restriction enzymes to digest genomic DNA and construct a reduced represented library (RRL) of genomes, which are then multiplexed into one library for NGS using DNA barcoded adapters (Elshire et al. 2011; Poland et al. 2012). Researchers have adopted the GBS approach to successfully detect SNP markers and construct genetic linkage maps containing thousands of SNP markers for QTL studies in many plant and animal species, including wheat, barley, lettuce, pea, tomato, peach, drosophila, and foxes (Poland et al. 2012; Truco et al. 2013; Johnson et al. 2015; Boutet et al. 2016; Hackett et al. 2016; Celik et al. 2017; Gao et al. 2019; Gonda et al. 2019). In the current study, we have 1) identified over 8000 SNP markers polymorphic between PI 270443 and NC EBR- 2, 2) constructed a high-density genomic-bin linkage map of the new RIL population, 3) discovered and fine mapped QTLs for LB resistance conferred by PI 270443, and 4) identified candidate resistance related genes in the QTLs’ peak locations for future investigations.

## Materials and method

### Plant material

Previously, we developed an interspecific recombinant inbred line (RIL) population of tomato (*n* = 166 lines) from a cross between a LB-susceptible *Solanum lycopersicum* breeding line NC EBR- 2 and the LB-resistant *S. pimpinellifolium* accession PI 270443, and used it to estimate the heritability of LB resistance in the RIL population (Jia and Foolad 2019). The advanced tomato breeding line NC EBR- 2 has numerous desirable horticultural characteristics, including large-size red fruit and resistance to early blight (caused by *Alternaria solani* and *A. tomatophila*) (Gardner 1988), but it is highly susceptible to LB. PI 270443 is a highly inbred line wild accession with indeterminate growth habit and prolific small shiny red fruit, which we previously determined to be highly resistant to LB (Foolad et al. 2014a). In the present study, we used 122 lines of the F_9_ and F_10_ generations of the RIL population for genetic map construction and discovery of QTLs conferring LB resistance in PI 270443. In all disease screening experiments of the RIL population, LB-susceptible and LB-resistant control lines, including Fla. 8059 (highly susceptible), New Yorker (highly susceptible, containing *Ph- 1*), NC 63 EB (partial resistant, *Ph- 2*), NC 870 (good resistant, *Ph- 3*), and NC 03220 (highly resistant, *Ph- 2* + *Ph- 3*), were included. The original seed of PI 270443 was received from USDA ARS Plant Genetic Resources Unit (PRGU), Geneva, NY, USA, seeds of New Yorker and all NC lines were provided by R.G. Gardner, North Carolina State University, Miles River, NC, USA, and seed of Fla.8059 was obtained from J.W. Scott, University of Florida, Gulf Coast Research & Education Center, Wimauma, FL, USA.

### Disease screening in the F_9_ and F_10_ generations

For each of the F_9_ and F_10_ generations of the RIL population (*n* = 122) two LB-screening experiments were conducted, including F_9_ Exp. I, F_9_ Exp. II, F_10_ Exp. I, and F_10_ Exp. II. In each disease screening experiment, six seedlings from each RIL were grown to 6-week-old and separated into two replicates in an environmentally-controlled greenhouse (GH) at The Pennsylvania State University, University Park campus.. In all experiments, the parental lines of the RIL population (PI 270443 and NC EBR- 2), F_1_ generation, and several control genotypes were also included. Inoculum preparation and plant inoculation with *P. infestans* isolates were as described elsewhere (Jia and Foolad 2019). Briefly, a blend of 3 local *P. infestans* isolates (A1 mating type, US- 23 clonal lineage) were used. Six-week-old healthy tomato seedlings were primed for inoculation in dark, cool (16–20 °C) and humid (> 90% relative humidity, RH) GH conditions for 7 h. An inoculum suspension (~ 8000 sporangia/mL) was directly sprayed onto the seedlings twice, with a 30-min interval. The average temperature and RH during the experiments were 16–20 °C and 80–90%, respectively. Approximately after 7–10 days post-inoculation (depending on experiment conditions) LB disease severity (DS) was visually estimated for each plant based on foliage symptoms. The percentage DS (%DS) ranged from 0% (no symptom) to 100%(total defoliation). Example images of %DS of parental lines and a few RILs are shown in Fig. 3. Each RIL’s %DS was averaged over six individual plants (six biological replicates).

### Tissue collection and DNA isolation

Prior to disease inoculation, leaf tissue was collected individually from 4-week-old seedlings of all 166 F_10_ RILs, and parental and F_1_ plants. Leaf tissues were frozen immediately using liquid nitrogen and stored at − 80 °C. Total genomic DNA was isolated from 100 mg leaf tissue samples with a modified CTAB protocol, using a commercial CTAB buffer and silica spin column (OPS Diagnostics). RNase was added to samples to avoid RNA contamination in the DNA. Genomic DNA was quantified with a Cytation™ 3 Multi-Mode Reader (BioTek® Instruments, Inc.). Randomly selected genomic DNA samples were run on 0.8% agarose-water gel to ensure the presence of high molecular weight intact genomic DNA.

### GBS library preparation and illumina sequencing

A combination of a “rare-cutter” (*TseI*) and a “common-cutter” (*CviAII*) restriction enzymes was chosen for the two-enzyme GBS analysis. Following restriction enzyme digestion, DNA fragments were ligated to adapters with 8-bp buffer sequence and 6–9 bp sample-specific barcodes developed by Dr. Bode Olukolu (Wadl et al. 2018). Two 96-plex plates were pooled into two separate libraries, size selected, and PCR amplified following a unique protocol developed at NC State (Wadl et al. 2018). Both libraries were subsequently sequenced for 125 bp single-end using *Illumina HiSeq 2500* (two lanes) at NC State Genomic Sciences Laboratory, Raleigh, NC, USA.

### Demultiplexing and SNP detection

The *FASTQ* files from Illumina sequencing were downloaded, and quality control was performed using *FastQC* (Andrews 2010). The 8-bp buffer sequences were trimmed using *FASTX-trimmer* (Hannon 2010), and barcode demultiplexing was conducted with Sabre (https://github.com/najoshi/sabre). The demultiplexed reads were mapped onto the tomato genome SL3.0 (International Tomato Genome Sequencing Project 2017) using *BWA-mem* (Li and Durbin 2009), and the alignment rate of each file was verified using *SAMtools* (Li et al. 2009). The parental alignment files of NC EBR- 2 and PI 270443 (BAM files) were merged respectively, and BAM files of all samples were processed using *Picard Tookit* (Broad Institute 2019) in preparation for SNP discovery. SNPs between parental lines were identified using the software *GATK* (McKenna et al. 2010) with *HaplotypeCaller* and genotyping mode *DISCOVERY*. The resulting parental variant call format (VCF) file was filtered to remove InDels, and AF (allele frequency) of 0.5 and AN (allele number) of 4 were used for filtering valid parental SNPs. Another method was also employed to identify additional parental SNPs, using *SAMtools v. 0.1.7a* and custom Perl scripts previously published (Hulse-Kemp et al. 2016), and new SNPs were filtered using the same criteria. The two SNP sets between the parents were merged, and a non-redundant set of SNPs was created.

The combined parental SNP file was used to call SNPs for the RIL population using *GATK-HaplotypeCaller* and genotyping mode *GENOTYPE_GIVEN_ALLELES* with output mode *EMIT_ALL_SITES*. The resulting VCF file was first filtered (Quality > 30, Minor allele frequency > 0.1 and < 0.9, samples with genotype data > 80%) using *SnpSift* (http://snpeff.sourceforge.net/SnpSift.html) filter option. Individual RILs with high levels of missing data were filtered out using *VCFtools* (Danecek et al. 2011), resulting in 130 RILs. The final VCF file containing SNPs detected among the RILs was converted into ABH mapping data format, using a custom Perl script developed by Gonda et al. (Gonda et al. 2019), including “A” as the genotype of NC EBR- 2, “B” as the genotype of PI 270443, and “H” as the heterozygous.

### Genomic bins and genetic map construction

The ABH-type genotype data of the RIL population was pre-processed in Excel by changing heterozygous loci (“H”) to missing information (“-”). Some missing data were filled by using information from the flanking marker genotypes. For instance, if most genotypes showed “AAAAAAAA” in one genomic region and one sample contained a missing nucleotide in that region, e.g., “AAAA-AAA”, the missing nucleotide was manually filled with an “A”. The processed AB- genotype data file was uploaded onto the JoinMap®5 program (van Ooijen 2019), and 8 more individuals were eliminated due to excessive heterozygosity. To construct a genetic linkage map, the maximum likelihood estimation (MLE) mapping, with a minimum independence LOD score of 4, was employed to determine linkage groups, marker orders, and recombination frequencies. The default MLE mapping parameters in JoinMap®5 were used. Marker segregation patterns were examined in JoinMap®5 using a chi-square goodness-of-fit test (*p* < 0.01). Subsequently, the"*exclude identical marker*"option was employed to group markers with zero recombination between them into genomic bins, and a genetic linkage bin map with 1,195 genomic bins was constructed. MapChart v2.3 software (Voorrips 2002) was employed to create images of the genetic map.

### High resolution QTL analysis

The 1,195-SNP genomic bin map and LB %DS scores from all 4 RIL experiments (F_9_ and F_10_ generations) were used for QTL mapping, by employing WinQTL Cartographer with single-trait composite interval mapping (Nascimento et al. 2001) and 1000 iterations of permutation tests (Wang et al. 2006).

## Results

### GBS and genetic linkage maps

Genotyping-by-sequencing (GBS) analysis of the original RIL population (*n* = 166 lines) and its two parental lines produced a total of 482,456,148 reads, achieving a genome coverage of 28.5X based on the tomato reference genome SL3.0 (International Tomato Genome Sequencing Project 2017). Specifically, the genome coverages were 3X for the parental line NC EBR- 2, 4X for the parental line PI 270443, and 0.29X (on average) for the RI lines (ranging from 0.001X to 0.7X).

Alignment of the demultiplexed files to tomato reference genome SL3.0 resulted in high alignment rates, over 99.5% for most of the RILs. Only 36 RILs had alignment rates less than 99.5%, correlating with lower genome coverages in those RILs. Initially, a total of 153,096 variants were called using the parental alignment files (BAM); the variant call format (VCF) was filtered to remove InDels, resulting in 34,498 variants. Further filtering (AF = 0.5, AN = 4) ensured the variants were valid SNPs between NC EBR- 2 and PI 270443, yielding 20,359 SNPs. Another pipeline with *SAMtools* (v. 0.1.7a) and custom *Perl* scripts was used to identify an additional 1,851 unique parental SNPs (Hulse-Kemp et al. 2016). Thus, 22,210 parental SNPs were used to call genotypes in the RIL population. Filtering out RILs with low alignment rates and SNPs with > 20% missing data (AN > 212) resulted in 8,553 SNPs among 130 RILs for the genetic map construction.

During map construction, we further eliminated 8 RILs due to excessive heterozygosity and 83 SNPs due to improper marker placement. Therefore, the high-density genetic linkage map was constructed based on using 8,470 SNPs and 122 RILs. Genetic binning of SNP markers resulted in 1,195 marker bins (each containing 1—1183 SNPs) and a genetic bin map (Suppl. Figure 1) encompassing a total genetic distance of 1,234.5 cM, which was similar to the total genetic distance of 1231.8 cM based on the 8,470 SNP markers (Table 1).

**Table 1:** Genetic distance by chromosome for the 1,195 SNP-based genetic bin map and 8,470 SNP-based genetic map and recombination count per RI line

Chr Number	Genetic Distance (cM)		Recombination count per RI line	
	8,470 SNPs Map	1,195 SNPs Bin Map	Average	Range
ch1	132.2	132.2	4.93	0–12.1
ch2	100.0	101.3	3.79	0–10
ch3	131.0	130.5	4.90	0–14
ch4	107.5	107.5	4.05	0–12
ch5	94.0	94.0	3.47	0–10
ch6	86.0	86.9	3.29	0–10
ch7	105.6	105.6	3.73	0–10
ch8	90.5	90.5	3.41	0–10
ch9	107.0	107.0	3.96	0–12
ch10	78.1	78.1	2.90	0–12
ch11	97.2	97.2	3.65	0–10
ch12	102.7	103.7	3.80	0–12.2
**Total**	**1231.8 cM**	**1234.5 cM**	**45.89**	**24.3–89.4**

**Fig. 1:**
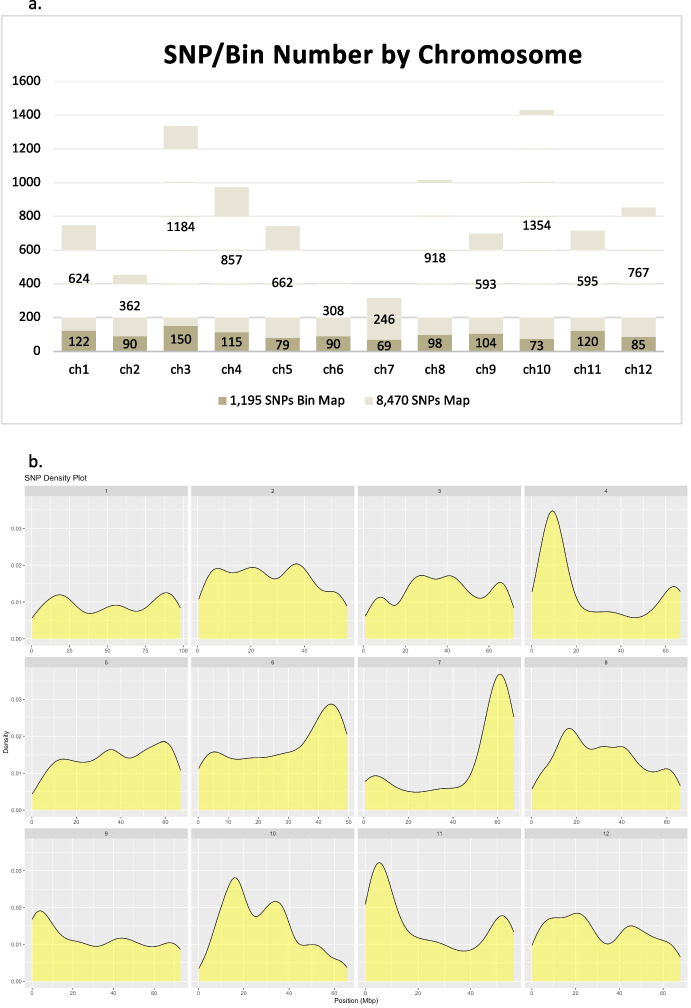
(**a**) Number of SNPs by chromosome used for the 1,195-SNP based genetic bin map and 8,470-SNP based genetic map. (**b**). SNP distribution by physical location for the total of 8,470 SNPs across 12 chromosomes

**Fig. 2:**
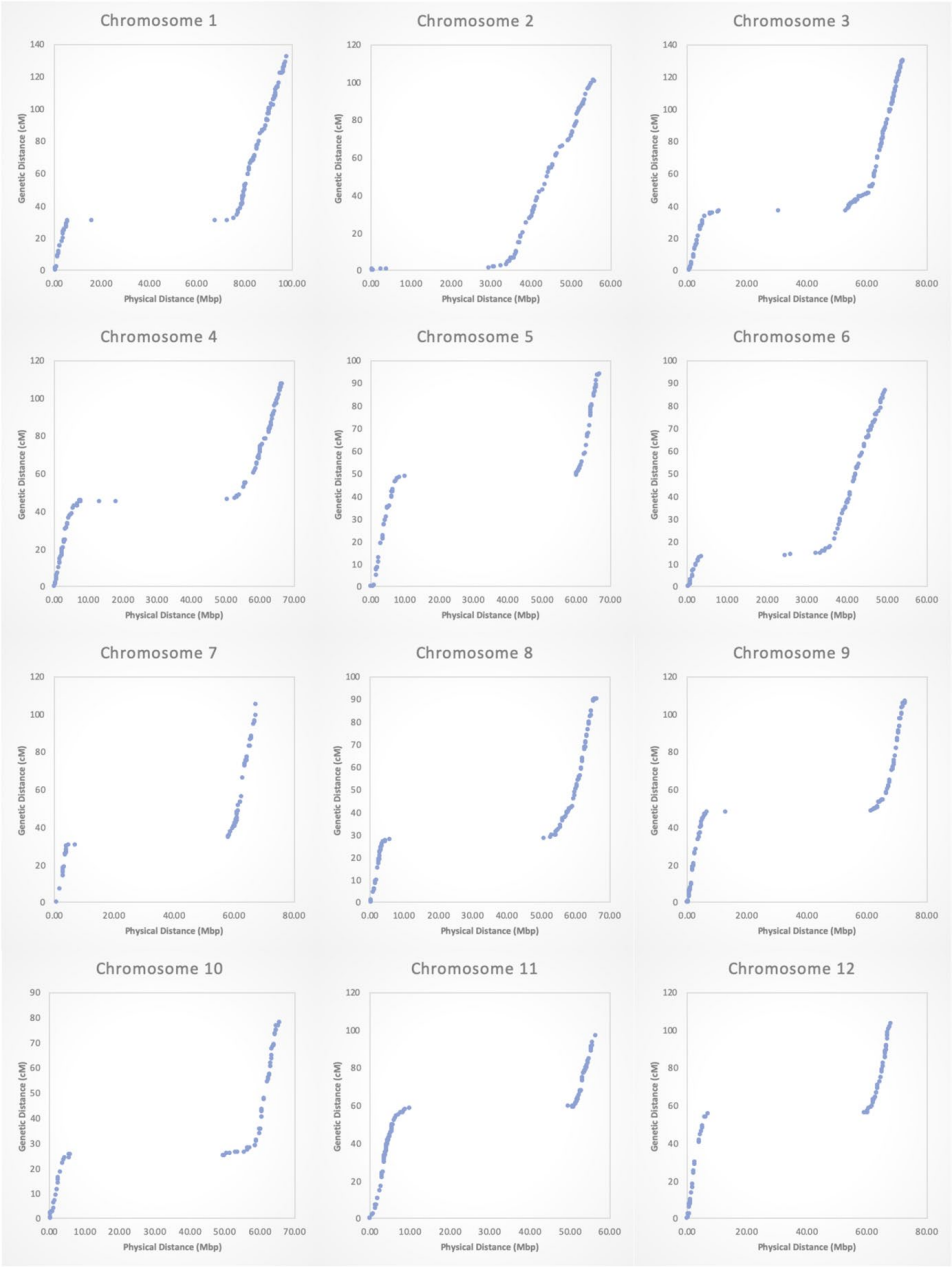
Relationship between physical and genetic locations within each chromosome. Each blue dot indicates genetic and physical location of a representative SNP marker for each genomic recombinant bin of the bin map

The number of SNP markers per chromosome for both the 1,195-bin map and 8,470-SNP-map are shown in Fig. 1a. For the SNPs map, the number of markers per chromosome ranged from 246 (chr 7) to 1,354 (chr 10), with an average of ~ 706 SNPs per chromosome; for the bins map, the number of bins per chromosome ranged from 69 (chr 7) to 150 (chr 3), with an average of ~ 100 bins per chromosome (Fig. 1a). All SNP markers distributed evenly across each of the 12 chromosomes (Fig. 1b). On average, there was 0.96 recombinant bin per cM of genetic distance. Chromosome 10 was the shortest (78.1 cM) and chromosome 1 the longest (132.2 cM) in both genetic linkage maps (SNPs and bins; Table 1). The estimated average number of meiotic recombination per RIL for each chromosome, as determined by JoinMap®5 using 1,195 binned SNP markers, was proportional to the chromosome size, ranging from 2.90 recombination events for chromosome 10 (to 4.93 for chromosome 1 (Table 1). Further, each individual RIL varied for the estimated total recombination counts across the 12 chromosomes, ranging from 24.3 to 89.4, with an average of 45.9 recombination events (Table 1). When comparing the genetic bin map (based on 1,195 SNP markers) with the physical map of the SNP markers (SL3.0), it appeared that high recombination events occurred on the interstitial and distal (telomeric) ends of all 12 tomato chromosomes, while recombination events were significantly depressed in the pericentric regions of the chromosomes (Fig. 2). Furthermore, all 12 chromosomes exhibited similar recombination rates with no meiotic recombination repression outside of the pericentric regions.

### Genetic composition of the RILs and marker segregation

The genetic composition of the 122 F_10_ RILs used for linkage map construction included 50.8% homozygous alleles from the cultivated parent (NC EBR- 2), 47.4% homozygous alleles from the wild parent (PI 270443), and 1.8% heterozygous markers (which were counted as missing data). Seven genomic regions, on chromosomes 1, 2, 3, 8, 10 and 11, exhibited significant (*p* < 0.01) skewed segregations (Suppl. Table 1). Of these, while 2 regions, including one on chromosome 10 (53.8 cM, 61 Mbp) and one on chromosome 2 (17.6 cM, 4.2 Mbp) exhibited strong skewness in favor of the wild parent PI 270443 (64%—75% PI alleles), 5 regions, on chromosomes 1, 3, 8 and 11, showed moderate skewness in favor of the cultivated parent NC EBR- 2 (~ 60% NC alleles; Suppl. Table 1). The extent of segregation distortion in this RIL population was generally similar to that observed in an F_2_ population of the same cross (Merk et al. 2012). There were no clear relationships between the chromosomal locations of skewed segregations and locations of the identified QTLs; while segregation distortions were observed on chromosomes 1, 2, 3, 8, 10 and 11, QTLs were identified on chromosomes 1, 10 and 12 (described below).

### Disease performance of the different genotypes

**Fig. 3:**
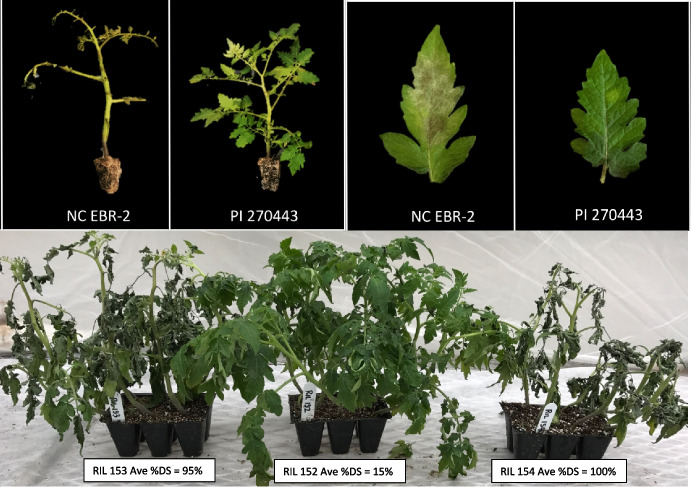
Example images of parental lines (NC EBR- 2, susceptible; and PI 270443, resistant) and RILs, which were grown in the same tray, after disease screening experiments

The disease performances of the parental lines (LB-susceptible NC EBR- 2, and LB-resistant PI 270443), F_1_ generation, and 5 control genotypes, Fla. 8059 (susceptible), New Yorker (susceptible, containing *Ph- 1*), NC 63 EB (resistant, containing *Ph- 2*), NC 870 (resistant, containing *Ph- 3*) and NC 03220 (resistant, containing *Ph- 2* + *Ph- 3*), were previously reported elsewhere (Jia and Foolad 2019). Briefly, NC EBR- 2 exhibited high %DS across all 4 experiments, ranging from 90 to 100% with an average of 98.7% (Fig. 3), statistically similar to the susceptible control lines Fla. 8059 (%DS = 99.3%) and New Yorker (%DS = 97.4%); PI 270443 showed very strong resistance to LB, with %DS ranging from 0 to 15% with an average of 4.5% (Fig. 3), statistically exceeding the resistance levels in the resistant lines NC 03220 (average %DS = 15.0%), NC 870 (average %DS = 33.6%) and NC 63 EB (average %DS = 46.3%); the F_1_ progeny showed moderate resistance to LB across all experiments, with an average %DS = 23.4%, statistically similar to the resistant line NC 03220 (Jia and Foolad 2019).

**Fig. 4:**
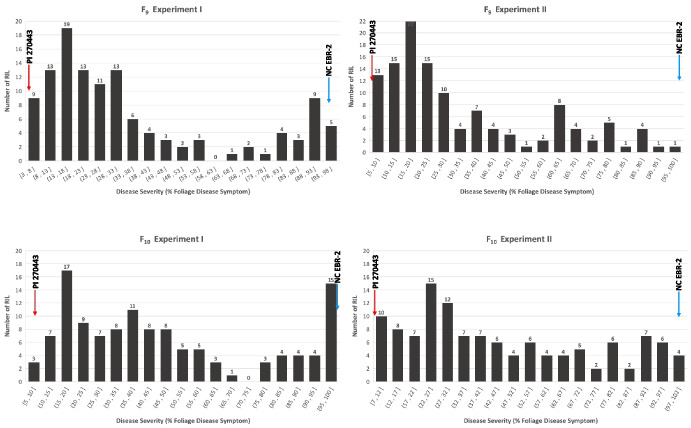
Frequency distribution of late blight disease severity in 122 F_9_ and F_10_ RI lines in Experiment I and II. Disease severity is measured as the percentage foliage disease symptom (% DS) of whole plants caused by late blight infection under greenhouse conditions

The RIL population was evaluated for response to LB disease in 4 screening experiments, including 2 experiments in F_9_ generation and 2 experiments in F_10_ generation. In F_9_ Exp. I, the %DS of the RILs (*n* = 121) ranged between 2.8% and 96.5%, with an average of 35.8% (Table 2, Fig. 4), and in F_9_ Exp. II, the %DS of the RILs (*n* = 122) ranged between 5.3% and 100%, with an average of 34.0% (Table 2, Fig. 4). In both F_9_ experiments, the disease responses of the RIL population were continuous but not normally distributed, as determined by Shapiro–Wilk test of normality (*p* < 0.001); the distributions were heavily skewed towards resistance with significant skewness of 1.008 and 0.982 in experiments I and II, respectively (Fig. 4). In F_10_ Exp. I, the %DS of the RILs (*n* = 122) ranged between 5.2% and 100%, with an average of 47.2% (Table 2, Fig. 4), and in F_10_ Exp. II, the %DS of RILs (*n* = 122) ranged between 6.8% and 100%, with an average of 46.2% (Table 2, Fig. 4). Similar to that in the F_9_ experiments, in both F_10_ experiments the disease responses of the RIL population were continuous and not normally distributed, with significant (*p* < 0.001) skewness towards resistance, with skewness values of 0.613 and 0.507 for experiments I and II, respectively (Fig. 4). There were significant positive correlations between the %DS of RI lines across all 4 screening experiments (Table 3).

**Table 2:** Average late blight (LB) disease severity^1^ (% disease severity, % DS ± SE) for the F_9_ and F_10_ generations of the RIL population evaluated under controlled greenhouse conditions

		Final % DS^1^	
Generation	Number of lines	Average	Range of means
RIL F_9_ Exp. I	121	35.8 ± 2.6	2.8–96.5
RIL F_9_ Exp. II	122	34 ± 2.2	5.3–100
RIL F_10_ Exp. I	122	47.2 ± 2.7	5.2–100
RIL F_10_ Exp. II	122	46.2 ± 2.5	6.8–100

^1^ The percentage disease severity (% DS) is measured as percentage disease symptom of whole plants caused by P. infestans infection

**Table 3:** Correlation analysis among four disease response experiments conducted in the F_9_ and F_10_ generations of the RIL population

	RIL F_9_ Exp II	RIL F_10_ Exp I	RIL F_10_ Exp II
RIL F_9_ Exp I	0.87	0.80	0.69
RIL F_9_ Exp II		0.81	0.78
RIL F_10_ Exp I			0.77

### QTL analyses

QTL analyses indicated the presence of 2 major QTLs for LB resistance on chromosome 10 of PI 270443, which were detected consistently in all 4 screening experiments. In addition, a few minor QTLs were identified in on chromosomes 1 and 12 of PI 270443, which were detected only in some of the experiments.

**Fig. 5:**
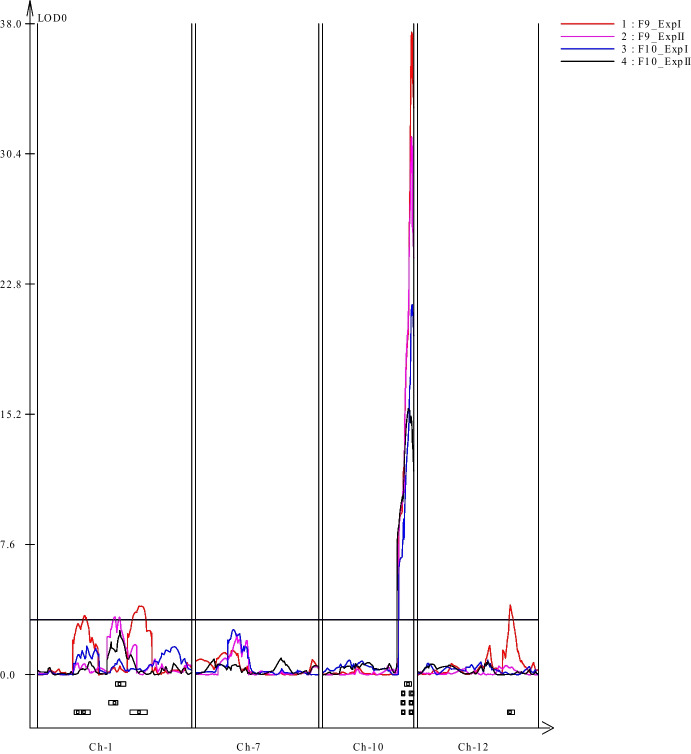
Late blight resistant QTLs detected in PI 270443 by WinQTL Cartographer v2.5. Data from all four experiments from the F_9_ and F_10_ generations are included in the QTL analyses, indicated by different line color as shown in figure legends. The X-axis represents chromosome number and relative location, while Y-axis represents LOD score generated by CIM mapping analysis. LBQTL- 1.1, LBQTL- 1.2, LBQTL- 1.3 shown in Ch- 1, and LBQTL- 10.1, LBQTL- 10.2 shown in Ch- 10, and LBQTL- 12 shown in Ch- 12

At the QTL location on chromosome 10, two adjoining QTL regions were detected, which were named LBRQTL- 10.1 and LBRQTL- 10.2. The range of the LBRQTL- 10.1 was predominantly predicted at 65–70 cM (*SL3.0ch10 - 63231159* – *SL3.0ch10 - 63907108*) and peaking around 69 cM, with the exception of F_10_ Experiment II data where it spanned 64–74 cM (*SL3.0ch10 - 63185641* – *SL3.0ch10 - 64418420*) and peaking around 73 cM (Table 4, Fig. 5); the estimated average PVE for LBRQTL- 10.1 was 31%. The LBRQTL- 10.2 was predicted at 70–78 cM (*SL3.0ch10 - 63907108* – *SL3.0ch10 - 65525731*) and peaking around 76 cM in all experiments (Table 4, Fig. 5), with the estimated average PVE of 53%.

**Table 4:** QTLs detected for LB resistance based on CIM in F_9_ and F_10_ generations of the RIL population using WinQTL Cartographer V2.5; Major QTLs are shown in bold

QTL Name	Chr #	Phenotype Data	Genetic position (cM)	Markers Interval	Peak LOD*	Add	R^2^
**LBRQTL- 10.1**	10	F_9__ExpI	65.1–69.6	SL3.0ch10_63231159 – SL3.0ch10_63907108	12.12	17.86	0.33
		F_9__ExpII	65.1–69.6	SL3.0ch10_63231159 – SL3.0ch10_63907108	11.50	14.52	0.29
		F_10__ExpI	65.1–69.6	SL3.0ch10_63231159 – SL3.0ch10_63907108	9.11	15.68	0.24
		F_10__ExpII	63.8–74.4	SL3.0ch10_63185641 – SL3.0ch10_64418420	15.56	19.72	0.37
**LBRQTL- 10.2**	10	F_9__ExpI	70.1–77.8	SL3.0ch10_63907108 – SL3.0ch10_65525731	37.55	28.29	0.70
		F_9__ExpII	70.1–77.8	SL3.0ch10_63907108 – SL3.0ch10_65525731	31.45	23.65	0.57
		F_10__ExpI	70.1–77.8	SL3.0ch10_63907108 – SL3.0ch10_65525731	21.64	24.57	0.46
		F_10__ExpII	74.9–77.8	SL3.0ch10_64418420 – SL3.0ch10_65525731	15.10	21.06	0.38
LBRQTL- 1.1	1	F_9__ExpI	39–41.7	SL3.0ch01_78453163 – SL3.0ch01_78947444	3.46	5.65	0.04
LBRQTL- 1.2	1	F_9__ExpII	64.9–70.5	SL3.0ch01_82077831 – SL3.0ch01_84336581	3.37	4.78	0.04
LBRQTL- 1.3	1	F_9__ExpI	81.3–92.3	SL3.0ch01_86059739 – SL3.0ch01_89093242	4.02	5.98	0.04
LBRQTL- 12	12	F_9__ExpI	78.3–81.6	SL3.0ch12_64819062 – SL3.0ch12_65159849	4.07	6.54	0.04

^*^ LOD: log of likelihood; Add: additive effects, positive effects inherited from PI 270443 parent and negative effects from NC EBR- 2 parent: R^2^: represents percent phenotypic variation explained by the corresponding QTL. Experiments with no QTL detected are not shown in the table

In addition to LBRQTL- 10.1 and LBRQTL- 10.2, four minor putative LB-resistance QTLs were detected in PI 270443, three located on chromosome 1 (LBRQTL- 1.1, LBRQTL- 1.2 and LBRQTL- 1.3) and one located on chromosome 12 (LBRQTL- 12); the PVE for each of these QTLs ranged from 3.5% to 4.0% (Table 4; Fig. 5).

### Disease resistance related genes in the QTL regions

We extracted gene models from iTAG3.2 for all the LBRQTL regions. For the major LB-resistance QTLs identified on chromosome 10, the LBRQTL- 10.1 and LBRQTL- 10.2 regions encompassed 94 and 216 genes, respectively. Based on a literature review of the LB-resistance genes in tomato and potato, as well as general disease resistance genes in tomato (see Discussion), a total of 18 “disease-related” candidate genes were identified in this region on chromosome 10 (Suppl. Table 2). For the 3 minor LB-resistance QTLs identified on chromosome 1, the LBRQTL- 1.1, LBRQTL- 1.2, and LBRQTL- 1.3 encompassed 40, 262, and 421 genes, respectively. Within these 3 locations, a total of 17 “disease-related” candidate genes were identified (Suppl. Table 2). The minor LB-resistance QTL identified on chromosome 12 (LBRQTL- 12) encompassed 57 genes, of which 2 were deemed to be related to disease resistance (Suppl. Table 2).

## Discussion

New and more destructive genotypes of *P. infestans* have been reported in tomato fields in recent years in the United States (USAblight.org 2019; Pieterse 2018); such genotypes may overcome resistance in the current LB-resistant cultivars under disease favorable conditions. It is crucial to identify and genetically characterize new sources of LB resistance and incorporate additional resistance genes/QTLs into new tomato cultivars. In multiple studies and across numerous replicated experiments, *S. pimpinellifolium* accession PI 270443 exhibited superior LB resistance, where its %DS was significantly lower than that of the LB-resistant control lines containing *Ph- 2*, *Ph- 3*, or *Ph- 2* + *Ph- 3* combined in homozygous conditions (Foolad et al. 2014b; Merk et al. 2012; Merk and Foolad 2012). This resistance superiority along with its high heritability (Jia and Foolad 2019) suggest the considerable value of PI 270443 as a new source of LB resistance for tomato breeding.

To understand genetic basis of LB resistance in PI 270443, we previously developed a RIL population (*n* = 166) from a cross between this accession and LB-susceptible tomato breeding line NC EBR- 2, and investigated the inheritance of resistance using F_9_ and F_10_ generations of the RILs (Jia and Foolad 2019). The moderately high estimate of *h*^*2*^ (0.76) suggested that the underlying genetics of the LB-resistance in PI 270443 was rather simple, and potentially controlled by a few resistance genes (Jia and Foolad 2019). To further investigate the genetic basis of LB-resistance in PI 270443, a subset of the RIL population (*n* = 122) was employed for genetic mapping and identification and characterization of QTLs underlying LB resistance in this accession. Both F_9_ and F_10_ generations were used for disease screening and QTL mapping. A generally higher average %DS was observed in the F_10_, compared to the F_9_, generation (Table 2), which most likely was due to variations in the environmental conditions of the GH experiments, including disease pressure, lighting and RH. Further, in all 4 disease screening experiments of the RIL population, disease distributions were continuous, though approaching bimodal and skewing towards resistance (Fig. 4). The latter observations may indicate a qualitative nature of LB resistance in PI 270443, consistent with the moderately high estimates of *h*^*2*^ observed in our previous study (Jia and Foolad 2019).

We undertook a GBS approach to simultaneously sequence the parental lines and genotype the F_10_ RIL population, using the restriction enzyme combination of *TseI/CviAII*. This two-enzyme GBS system employing a rare-cutting and a frequent-cutting restriction enzyme was chosen because it simplified the process of library quantification before sequencing (Poland et al. 2012). In silico digestion of tomato genome with this enzyme combination yielded 327,375 fragments between 100–350 bp, suggesting that the enzymes were capable of digesting tomato genomes evenly, suitable for the construction of a high-quality RRL. An ultra-high-density genetic linkage map of the RIL population with 8,470 SNP markers was developed, showing a total genetic map length of 1231.8 cM (Table 1). The genetic length and chromosomes’ centromeric regions of this map (Fig. 2, Table 1, Suppl. Figure 1) agreed with those of other recently reported genetic maps of tomato (Sim et al. 2012; Gonda et al. 2019; Ohlson et al. 2018). To facilitate and simplify QTL mapping, SNPs were further binned into 1,195 genomic bins, resulting in a high-density genetic bin map with a total genetic distance of 1234.5 cM (Table 1) and a total physical distance of 802.8 Mbp. Across chromosomes, the average distance between linked genomic bins was ~ 1.0 cM, ranging from 0.0 cM (observed in a few chromosomes) to 10.6 cM (on chromosome 12, between SNP markers *SL3.0ch12 - 2588708* and *SL3.0ch12 - 4076338*).

The high-density genetic linkage map developed in the present study significantly improved our ability to identify and fine map QTLs underlying LB resistance in PI 270443, compared with the preliminary QTL mapping study conducted in an F_2_ population of the same cross using much smaller number of genetic markers (Merk et al. 2012); while the average marker spacing in the previous study was 7.2 cM, the average genetic distance between adjacent bins in the current study is ~ 1.0 cM. The previous study identified two genomic regions on chromosomes 1 and 10 of PI 270443 associated with LB resistance: *Ph- 5.1* was mapped on the long arm of chromosome 1 to a 23-cM interval between SSR markers SSRW11 and cTOE7 J7, and *Ph- 5.2* was mapped on the long arm of chromosome 10 to a 37.8-cM interval between SSR markers TMA0040 and SSR223 (Merk et al. 2012). The present study confirmed both QTL regions and further refined them to smaller intervals.

In the present study, a major LB-resistance QTL region was detected on chromosome 10 (Table 4, Suppl. Figure 1, Fig. 5), which overlapped with the QTL *Ph- 5.2* identified in our previous study (Merk et al. 2012). However, in the RIL population we detected 2 adjoining peaks in the same QTL region on chromosome 10: LBRQTL- 10.1, peaking at 69 cM and explaining ~ 31% PVE, and LBRQTL- 10.2, peaking at 76 cM and explaining ~ 53% PVE (Table 4, Suppl. Figure 1, Fig. 5). The two adjacent peaks detected in this region may indicate the presence of one major QTL for LB resistance, or two separate tightly-linked QTLs with the LBRQTL- 10.1 being a new one not previously reported. We also observed multiple disease-related candidate genes underlying the QTL region encompassing the two LOD peaks (discussed below).

Previous marker analysis indicated that accession PI 270443 did not contain the known and strong *Ph- 3* resistance gene on chromosome 9, but it suggested the presence of *Ph- 2* resistant gene on chromosome 10 of this accession (Foolad et al. 2014b). The most recent study confirmed that *Ph- 2* is the gene Solyc10 g085460 (RGA1) encoding a CC-NBS-LRR disease resistance protein on chromosome 10 (Pan et al. 2024), which is within the LBRQTL- 10.2 region at 64,748,798. There is a possibility that the resistance gene(s) underlying LBRQTL- 10.2 (detected in this study) and QTL *Ph- 5.2* detected earlier (Merk et al. 2012) are actually *Ph- 2*, or are in the same resistance-gene cluster as *Ph- 2*, since their physical locations do overlap. However, multiple GH and field LB disease evaluations have confirmed that the LB resistance expressed by PI 270443 is significantly stronger than the partial resistance conferred by *Ph- 2* alone (Merk et al. 2012; Foolad et al. 2014b; Jia and Foolad 2019) (also results from this study, unpublished data, and personal communications with R.G. Gardner at NC State), suggesting the presence of novel LB-resistance genes in PI 270443 other than *Ph- 2*. Further, the very large PVE by LBRQTL- 10.2 from the present study suggests the possibility of the presence of novel LB resistance genes in this region in PI 270443. In this region, other than the *Ph- 2* gene in tomato, a LB-resistance gene (*Rpi-ber*) has been mapped to 4.3 cM from the RFLP marker TG63 in the wild potato species *S. berthaultii* (Ewing et al. 2000). A genomic blast using the TG63 sequence (SGN-M88) confirmed the physical location of this marker on SL3.0 tomato reference genome between 64,891,355 bp and 64,891,857 bp, which is at 76.8 cM in our genetic bin map and near the peak LOD score for LBRQTL- 10.2 (76 cM); this observation suggests the possibility of homologous or homeologous relationships between the potato gene *Rpi-ber*, the tomato gene *Ph- 2* (Moreau et al. 1998; Zhi et al. 2021), and the gene(s) underlying LBRQTL- 10.2 in the present study. Further fine mapping and RNA-sequencing efforts are currently underway (at Penn State) to potentially identify resistance gene(s) underlying LBRQTL- 10.1 and LBRQTL- 10.2 in PI 270443.

Minor LB-resistance QTLs (with low LOD score and small PVE) were also detected in PI 270443, on chromosomes 1 (LBRQTL- 1.1, LBRQTL- 1.2 and LBRQTL- 1.3) and 12 (LBRQTL- 12); these QTLs were detected only in some experiments (Table 4). Our previous preliminary QTL mapping study using F_2_ generation of the same cross also detected a LB-resistance QTL on chromosome 1 (*Ph- 5.1*), between markers SSRW11 and cTOE7 J7 (Merk et al. 2012). In the present study, a genomic BLAST search, using PCR sequences previously used for SSRW11 and cTOE7 J7 marker genotyping, resulted in a physical genomic location of *Ph- 5.1* to be between 87,897,160 and 91,237,534 bp on tomato SL3.0 reference genome, and genetic map location of *Ph- 5.1* to be between 86–105 cM on the current genetic bin map. This region overlaps with LBRQTL- 1.3 (Table 4), which was detected in F_9_ Experiment I. For the minor LB-resistance QTL detected on the long arm of chromosome 12 of PI 270443 (peaking at 79.3 cM and 65,019,033 bp), no LB-resistance QTL was previously reported in *S. pimpinellifolium*; however, 2 LB-resistance QTLs (*lb12a, lb12b*) were previously reported in tomato wild species *S. habrochaites* on the short arm of chromosome 12 (Brouwer et al. 2004).

The high-density genetic linkage map constructed in the present study, together with the LB screening data from F_9_ and F_10_ generations of the RIL population, provided opportunities for a high-resolution genetic mapping, narrowing down the range and critical genomic locations of the LB-resistance QTLs identified on chromosomes 1 and 10 of PI 270443. We extracted gene models of the QTL regions from iTAG3.2 and, based on the literature review of LB-resistance genes in tomato and potato as well as general disease resistance genes in tomato, we identified 3 and 15 disease-related candidate genes from LBRQTL- 10.1 and LBRQTL- 10.2 regions, respectively, 17 disease-related candidate genes in the three QTLs on chromosome 1, and 2 disease related candidate genes in LBRQTL- 12 (Suppl. Table 2). Nearly 50 of the identified *Rpi* genes (for resistance to *P. infestans*) in Solanum species (all in potato except one) belong to the nucleotide-binding-site-leucine-rich repeat (NBS-LRR) gene family (Paluchowska et al. 2022), including the *Ph- 3* gene cloned in tomato (Zhang et al. 2014). In the present study, we identified 3 NBS-LRR candidate resistance genes in the major LB-resistance QTL region on chromosome 10 (Suppl. Table 2): one gene (Solyc10 g084085) encodes for a TIR-NBS-LRR gene and is located at 69 cM, which is the LOD peak for LBRQTL- 10.1, and 2 genes [Solyc10 g085460 (*Ph- 2*) and Solyc10 g085780] are located at 76 cM, which is the LOD peak for LBRQTL- 10.2 (Fig. 5, Suppl. Table 2). Additionally, 2 NBS-LRR genes (Solyc01 g088060, Solyc01 g088680) were identified in the minor QTL region LBRQTL- 1.2 on chromosome 1, which were within a 2-cM genetic distance from the LOD peak for the residing QTL (Fig. 5, Suppl. Table 2). These NBS-LRR genes are considered paramount candidate genes for future fine mapping studies.

We also investigated other potential resistance genes, including receptor-like protein/kinase (RLP/K), cysteine-rich transmembrane protein, glutathione S-transferase, WRKY transcription factors and pathogenesis-related proteins (Suppl. Tables 2), which co-localized with the LB-resistance QTLs on chromosomes 10, 1, and 12. It is possible that one or more of these genes underlie the LB-resistance QTLs identified in the present study. However, additional research, including RNA-seq based expression analyses and genetic transformation and/or gene editing, is necessary to examine potential contribution of these genes to the LB-resistance characteristics observed in PI 270443. Currently, further fine mapping of the identified QTLs is underway (at Penn State) using the RNA-sequencing method to identify candidate resistance gene(s) responsible for the strong LB resistance in PI 270443.

## Conclusion

The detailed QTL mapping efforts described in this study provide valuable information on SNP markers that are closely linked to the QTLs underlying LB resistance in PI 270443, extending the opportunity for breeders to utilize this accession and its derived RILs for transferring LB resistance into new tomato cultivars. At Penn State, we have transferred this resistance to our tomato breeding lines and experimental hybrids, which are being trialed for commercial release. Some of the derived hybrids carrying this source of resistance alone exhibit LB resistance comparable to cultivars with *Ph- 2* + *Ph- 3* combined, and when pyramided with *Ph- 2* and *Ph- 3* resistance genes, it has resulted in much stronger resistance than any existing LB resistant breeding line or hybrid cultivar. It should be noted, the RILs from this study, as well as breeding lines and experimental hybrids containing LB resistance from PI 270443 developed at Penn State, are available for research and breeding purposes upon signing MTAs with the Pennsylvania State University.

## Supplementary Information

Below is the link to the electronic supplementary material.

Supplementary file1 (DOCX 2133 KB)

Supplementary file2 (DOCX 17 KB)

Supplementary file3 (DOCX 20 KB)

## Data Availability

The raw reads of the GBS from 122 RILs and parental lines are available through NCBI short reads archive bioproject number PRJNA1099137. The genetic map and genotyping table are available via the Solanaceae Genome Network (SGN).

## Abbreviations

BAM: Binary alignment map
CAPS: Cleaved amplified polymorphic sequences
%DS: Percentage disease severity
ETI: Effector triggered immunity
GBS: Genotyping-by-sequencing
GH: Greenhouse
HR: Hypersensitive response
LB: Late blight
LBRQTL: Late-blight-resistance quantitative trait locus
LOD: Logarithm of odds
MAS: Marker assisted selection
MBA: Marker-based analysis
MLE: Maximum likelihood estimation
NBS-LRR: Nucleotide binding site and leucine-rich repeat
PAMP: Pathogen-associated molecular patterns
PCR: Polymerase chain reaction
PGRU: Plant Genetics Resources Unit
PPR: Pattern Recognition Receptors
PTI: PAMP-Triggered Immunity
PVE: Phenotypic variation explained
QTL: Quantitative trait locus
QTLs: Quantitative trait loci
RFLP: Restriction fragment length polymorphism
RH: Relative humidity
RLP/K: Receptor-like protein/kinase
ROS: Reactive oxygen species
RRL: Reduced represented library
SNP: Single nucleotide polymorphism
SSR: Simple sequence repeats
VCF: Variant call format

## References

[CR1] Andrews S (2010) FastQC: a quality control tool for high throughput sequence data., 0.11.8 edn. Babraham Bioinformatics. http://www.bioinformatics.babraham.ac.uk/projects/fastqc

[CR2] Bhatia N, Tiwari JK, Kumari C, Zinta R, Sharma S, Buckseth T, Thakur AK, Singh RK, Kumar V (2024) Transcriptome analysis reveals genes associated with late blight resistance in potato. Sci Rep 14(1):15501. 10.1038/s41598-024-60608-3 38969681 10.1038/s41598-024-60608-3 PMC11226683

[CR3] Black LL, Wang TC, Hanson PM, Chen JT (1996) Late blight resistance in four wild tomato accessions: effectiveness in diverse locations and inheritance of resistance. J Phytopathol 86:S24

[CR4] Boutet G, Alves Carvalho S, Falque M, Peterlongo P, Lhuillier E, Bouchez O, Lavaud C, Pilet-Nayel M-L, Rivière N, Baranger A (2016) SNP discovery and genetic mapping using genotyping by sequencing of whole genome genomic DNA from a pea RIL population. BMC Genomics 17:121. 10.1186/s12864-016-2447-2 26892170 10.1186/s12864-016-2447-2 PMC4758021

[CR5] Broad Institute (2019) Picard toolkit. Broad Institute, GitHub repository. http://broadinstitute.github.io/picard/

[CR6] Brouwer DJ, St. Clair DA (2004) Fine mapping of three quantitative trait loci for late blight resistance in tomato using near isogenic lines (NILs) and sub-NILs. Theor Appl Genet 108(4):628–638. 10.1007/s00122-003-1469-8 14586504 10.1007/s00122-003-1469-8

[CR7] Brouwer DJ, Jones ES, St. Clair DA (2004) QTL analysis of quantitative resistance to *Phytophthora infestans* (late blight) in tomato and comparisons with potato. Genome 47(3):475–492. 10.1139/g04-001 15190365 10.1139/g04-001

[CR8] Celik I, Gurbuz N, Uncu AT, Frary A, Doganlar S (2017) Genome-wide SNP discovery and QTL mapping for fruit quality traits in inbred backcross lines (IBLs) of *Solanum pimpinellifolium* using genotyping by sequencing. BMC Genomics 18 (1). 10.1186/s12864-016-3406-7 10.1186/s12864-016-3406-7 PMC5209891 28049423

[CR9] Chen C-H, Sheu Z-M, Wang T-C (2008) Host specificity and tomato-related race composition of *Phytophthora infestans* isolates in Taiwan during 2004 and 2005. Plant Dis 92:751–755 30769600 10.1094/PDIS-92-5-0751

[CR10] Chen A-L, Liu C-Y, Chen C-H, Wang J-F, Liao Y-C, Chang C-H, Tsai M-H, Hwu K-K, Chen K-Y (2014) Reassessment of QTLs for late blight resistance in the tomato accession L3708 using a restriction site associated DNA (RAD) linkage map and highly aggressive isolates of *Phytophthora infestans*. PLoS ONE 9(5):e96417 24788810 10.1371/journal.pone.0096417 PMC4008630

[CR11] Chunwongse J, Chunwongse C, Black L, Hanson P (2002) Molecular mapping of the *Ph-3* gene for late blight resistance in tomato. J Horticult Sci Biotechnol 77(3):281–286. 10.1080/14620316.2002.11511493

[CR12] Cohen Y (2002) Populations of *Phytophthora infestans* in Israel underwent three major genetic changes during 1983 to 2000. Phytopathology 92(3):300–307 18944003 10.1094/PHYTO.2002.92.3.300

[CR13] Danecek P, Auton A, Abecasis G, Albers CA, Banks E, DePristo MA, Handsaker RE, Lunter G, Marth GT, Sherry ST, McVean G, Durbin R, Genomes Project Analysis G (2011) The variant call format and VCFtools. Bioinformatics 27(15):2156–2158. 10.1093/bioinformatics/btr330 21653522 10.1093/bioinformatics/btr330 PMC3137218

[CR14] Elsayed AY, da Silva HD, Mizubuti ESG, Carneiro CP (2011) Combing the monogenic and polygenic resistant genes to late blight in tomato. J Plant Breed Crop Sci 3(10):251–259

[CR15] Elshire RJ, Glaubitz JC, Sun Q, Poland JA, Kawamoto K, Buckler ES, Mitchell SE (2011) A robust, simple genotyping-by-sequencing (GBS) approach for high diversity species. PLoS ONE 6(5):e19379. 10.1371/journal.pone.0019379 21573248 10.1371/journal.pone.0019379 PMC3087801

[CR16] Ewing EE, Simko I, Smart CD, Bonierbale MW, May GD, Fry WE (2000) Genetic mapping from field tests of qualitative and quantitative resistance to *Phytophthora infestans* in a population derived from *Solanum tuberosum* and *Solanum berthaultii*. Mol Breeding 6:25–36. 10.1023/A:1009648408198

[CR17] FAOSTAT (2025) World tomato production in 2022. Food and Agriculture Organization of the United Nations. https://www.fao.org/faostat/en/#data/QV. Accessed 19 Jan 2025

[CR18] Foolad MR, Merk HL, Ashrafi H (2008) Genetics, genomics and breeding of late blight and early blight resistance in tomato. Crit Rev Plant Sci 27(2):75–107. 10.1080/07352680802147353

[CR19] Foolad MR, Sullenberger MT, Ohlson EW, Gugino BC (2014a) Response of accessions within tomato wild species, *Solanum pimpinellifolium* to late blight. Plant Breed 133:401–411. 10.1111/pbr.12172

[CR20] Foolad MR, Sullenberger MT, Ohlson EW, Gugino BK (2014b) Response of accessions within tomato wild species, *Solanum pimpinellifolium* to late blight. Plant Breeding 133(3):401–411. 10.1111/pbr.12172

[CR21] Foolad MR, Sullenberger MT, Ashrafi H (2015) Detached-leaflet evaluation of tomato germplasm for late blight resistance and its correspondence with field and greenhouse screenings. Plant Dis 99(5):718–722. 10.1094/PDIS-08-14-0794-RE 30699677 10.1094/PDIS-08-14-0794-RE

[CR22] Fry WE (2008) Phytophthora infestans: the plant (and R gene) destroyer. Mol Plant Pathol 9(3):385–402 18705878 10.1111/j.1364-3703.2007.00465.x PMC6640234

[CR23] Galindo J, Gallegly ME (1960) The nature of sexuality in *Phytophthora infestans*. Phytopathology 50(2):123–128

[CR24] Gallegly ME, Marvel ME (1955) Inheritance of resistance to tomato race-0 of *Phytophthora-infestans*. Phytopathology 45(2):103–109

[CR25] Gallegly ME (1960) Resistance to the late blight fungus in tomato. In: Proceedings of plant science seminar. Campell Soup Company, Camden, pp 113–135. https://www.cabidigitallibrary.org/doi/full/10.5555/19611601532

[CR26] Gao S, Foolad MR (2024) Identification and mapping of late blight resistance QTLs in the wild tomato accession PI 224710 (Solanum pimpinellifolium). Mol Breed 44(10):63. 10.1007/s11032-024-01498-1 39295771 10.1007/s11032-024-01498-1 PMC11405559

[CR27] Gao L, Gonda I, Sun H, Ma Q, Bao K, Tieman DM, Burzynski-Chang EA, Fish TL, Stromberg KA, Sacks GL, Thannhauser TW, Foolad MR, Diez MJ, Blanca J, Canizares J, Xu Y, van der Knaap E, Huang S, Klee HJ, Giovannoni JJ, Fei Z (2019) The tomato pan-genome uncovers new genes and a rare allele regulating fruit flavor. Nat Genet 51(6):1044–1051. 10.1038/s41588-019-0410-2 31086351 10.1038/s41588-019-0410-2

[CR28] Gardner RG (1988) NC EBR-1 and NC EBR-2 early blight resistant tomato breeding lines. HortScience 23:779–781

[CR29] Ghislain M, Byarugaba AA, Magembe E, Njoroge A, Rivera C, Roman ML, Tovar JC, Gamboa S, Forbes GA, Kreuze JF, Barekye A, Kiggundu A (2019) Stacking three late blight resistance genes from wild species directly into African highland potato varieties confers complete field resistance to local blight races. Plant Biotechnol J 17(6):1119–1129. 10.1111/pbi.13042 30467980 10.1111/pbi.13042 PMC6523587

[CR30] Gonda I, Ashrafi H, Lyon DA, Strickler SR, Hulse-Kemp AM, Ma Q, Sun H, Stoffel K, Powell AF, Futrell S, Thannhauser TW, Fei Z, Van Deynze AE, Mueller LA, Giovannoni JJ, Foolad MR (2019) Sequencing-based bin map construction of a tomato mapping population, facilitating high-resolution quantitative trait loci detection. Plant Genome 12 (1). 10.3835/plantgenome2018.02.0010 10.3835/plantgenome2018.02.0010 PMC12809921 30951101

[CR31] Hackett JL, Wang X, Smith BR, Macdonald SJ, Abney M, Palmer AA (2016) Mapping QTL contributing to variation in posterior lobe morphology between strains of *Drosophila melanogaster*. PLoS ONE 11(9):e0162573–e0162573. 10.1371/journal.pone.0162573 27606594 10.1371/journal.pone.0162573 PMC5015897

[CR32] Hannon GJ (2010) FASTX-Toolkit: FASTQ/A short-reads pre-processing tools. GitHub. https://github.com/agordon/fastx_toolkit

[CR33] Hulse-Kemp AM, Ashrafi H, Plieske J, Lemm J, Stoffel K, Hill T, Luerssen H, Pethiyagoda CL, Lawley CT, Ganal MW, Van Deynze A (2016) A HapMap leads to a *Capsicum annuum* SNP infinium array: a new tool for pepper breeding. Horticult Res 3:16036. 10.1038/hortres.2016.36 10.1038/hortres.2016.36 PMC4962762 27602231

[CR34] International tomato genome sequencing project (2017) Current tomato genome version SL3.0 and annotation ITAG3.10 https://solgenomics.net/organism/Solanum_lycopersicum/genome. Accessed 05/2018

[CR35] Jia M, Foolad MR (2019) Genetic analysis of late blight resistance in a new RIL population of tomato derived from LB-resistant *Solanum pimpinellifolium* accession PI 270443. Plant Breed 10.1111/pbr.12791

[CR36] Jo KR, Kim CJ, Kim SJ, Kim TY, Bergervoet M, Jongsma MA, Visser RG, Jacobsen E, Vossen JH (2014) Development of late blight resistant potatoes by cisgene stacking. BMC Biotechnol 14:50. 10.1186/1472-6750-14-50 24885731 10.1186/1472-6750-14-50 PMC4075930

[CR37] Johnson JL, Wittgenstein H, Mitchell SE, Hyma KE, Temnykh SV, Kharlamova AV, Gulevich RG, Vladimirova AV, Fong HWF, Acland GM, Trut LN, Kukekova AV (2015) Genotyping-By-Sequencing (GBS) detects genetic structure and confirms behavioral QTL in tame and aggressive foxes (*Vulpes vulpes*). PLoS ONE 10(6):e0127013–e0127013. 10.1371/journal.pone.0127013 26061395 10.1371/journal.pone.0127013 PMC4465646

[CR38] Jones JB, Zitter TA, Momol MT, Miller SA (2014) Compendium of tomato diseases and pests. Disease compendium series, 2nd edn. The American Phytopathological Society. https://books.google.com/books?id=YOUovwEACAAJ

[CR39] Li H, Durbin R (2009) Fast and accurate short read alignment with Burrows-Wheeler transform. Bioinformatics 25(14):1754–1760. 10.1093/bioinformatics/btp324 19451168 10.1093/bioinformatics/btp324 PMC2705234

[CR40] Li H, Handsaker B, Wysoker A, Fennell T, Ruan J, Homer N, Marth G, Abecasis G, Durbin R, Genome Project Data Processing S (2009) The sequence alignment/map format and SAMtools. Bioinformatics 25(16):2078–2079. 10.1093/bioinformatics/btp352 19505943 10.1093/bioinformatics/btp352 PMC2723002

[CR41] Li J, Liu L, Bai Y, Finkers R, Wang F, Du Y, Yang Y, Xie B, Visser R, van Heusden A (2011) Identification and mapping of quantitative resistance to late blight (*Phytophthora infestans*) in *Solanum habrochaites* LA1777. Euphytica 179(3):427–438. 10.1007/s10681-010-0340-7

[CR42] Li Q, Zhu H, Ai G, Yu J, Dou D (2024) Plant genes related to Phytophthora pathogens resistance. Phytopathol Res 6(1):15. 10.1186/s42483-024-00229-w

[CR43] Lough RC (2003) Inheritance of tomato late blight resistance in Lycopersicon hirsutum LA1033. North Carolina State University, Raleigh

[CR44] McGrath MT (2015) Late Blight Management in Tomato with Resistant Varieties. https://eorganic.org/node/10822. Accessed 17 May 2019

[CR45] McKenna A, Hanna M, Banks E, Sivachenko A, Cibulskis K, Kernytsky A, Garimella K, Altshuler D, Gabriel S, Daly M, DePristo MA (2010) The genome analysis toolkit: a MapReduce framework for analyzing next-generation DNA sequencing data. Genome Res 20(9):1297–1303. 10.1101/gr.107524.110 20644199 10.1101/gr.107524.110 PMC2928508

[CR46] Merk HL, Foolad MR (2012) Parent-offspring correlation estimate of heritability for late blight resistance conferred by an accession of the tomato wild species *Solanum pimpinellifolium*. Plant Breed 131(1):203–210. 10.1111/j.1439-0523.2011.01898.x

[CR47] Merk HL, Ashrafi H, Foolad MR (2012) Selective genotyping to identify late blight resistance genes in an accession of the tomato wild species Solanum pimpinellifolium. Euphytica 187(1):63–75. 10.1007/s10681-012-0729-6

[CR48] Moreau P, Thoquet P, Olivier J, Laterrot H, Grimsley N (1998) Genetic mapping of *Ph-2*, a single locus controlling partial resistance to *Phytophthora infestans* in tomato. Mol Plant Microb Interact 11(4):259–269. 10.1094/MPMI.1998.11.4.259

[CR49] Nascimento WM, Cantliffe DJ, Huber DJ (2001) Endo-beta-mannanase activity and seed germination of thermosensitive and thermotolerant lettuce genotypes in response to seed priming. Seed Sci Res 11:255–264

[CR50] Nowicki M, Foolad MR, Nowakowska M, Kozik EU (2012) Potato and tomato late blight caused by *Phytophthora infestans*: an overview of pathology and resistance breeding. Plant Dis 96(1):4–17. 10.1094/PDIS-05-11-0458 30731850 10.1094/PDIS-05-11-0458

[CR51] Ohlson EW, Ashrafi H, Foolad MR (2018) Identification and mapping of late blight resistance quantitative trait loci in tomato accession PI 163245. Plant Genome 11 (180007). 10.3835/plantgenome2018.01.0007 10.3835/plantgenome2018.01.0007 PMC12810023 30512045

[CR52] van Ooijen JW (2019) JoinMap®5 user manual: software for the calculation of genetic linkage maps in experimental populations of diploid species.

[CR53] Paluchowska P, Sliwka J, Yin Z (2022) Late blight resistance genes in potato breeding. Planta 255(6):127. 10.1007/s00425-022-03910-6 35576021 10.1007/s00425-022-03910-6 PMC9110483

[CR54] Pan C, Li X, Lu X, Hu J, Zhang C, Shi L, Zhu C, Guo Y, Wang X, Huang Z, Du Y, Liu L, Li J (2024) Identification and functional analysis of the Ph-2 gene conferring resistance to late blight (Phytophthora infestans) in Tomato. Plants (Basel) 13 (24). 10.3390/plants13243572 10.3390/plants13243572 PMC11679936 39771270

[CR55] Peirce LC (1971) Linkage tests with *Ph* conditioning resistance to race 0, *Phytophthora infestans*. Rep Tomato Genet Coop 21:30

[CR56] Pieterse L (2018) US scientists identified new mefenoxam resistant late blight strain. Potato News Today. https://potatonewstoday.com/2018/08/27/us-scientists-identified-new-mefenoxam-resistant-late-blight-strain/. Accessed 17 May 2019

[CR57] Poland JA, Brown PJ, Sorrells ME, Jannink JL (2012) Development of high-density genetic maps for barley and wheat using a novel two-enzyme genotyping-by-sequencing approach. PLoS ONE 7 (2). 10.1371/journal.pone.0032253 10.1371/journal.pone.0032253 PMC3289635 22389690

[CR58] Rodewald J, Trognitz B (2013) Solanum resistance genes against *Phytophthora infestans* and their corresponding avirulence genes. Mol Plant Pathol 14(7):740–757. 10.1111/mpp.12036 23710878 10.1111/mpp.12036 PMC6638693

[CR59] Sim S-C, Durstewitz G, Plieske J, Wieseke R, Ganal MW, Van Deynze A, Hamilton JP, Buell CR, Causse M, Wijeratne S, Francis DM (2012) Development of a large SNP genotyping array and generation of high-density genetic maps in tomato. PLoS ONE 7(7):e40563. 10.1371/journal.pone.0040563 22802968 10.1371/journal.pone.0040563 PMC3393668

[CR60] Smoot JJ, Gough FJ, Lamey HA, Eichenmuller JJ, Gallegly ME (1958) Production and germination of oospores of *Phytophthora infestans*. Phytopathology 48(3):165–171

[CR61] Sullenberger MT, Jia M, Gao S, Ashrafi H, Foolad MR (2022) Identification of late blight resistance quantitative trait loci in Solanum pimpinellifolium accession PI 270441. Plant Genome 15(4):e20251. 10.1002/tpg2.20251 35962567 10.1002/tpg2.20251 PMC12807060

[CR62] Sullenberger MT, Foolad MR (2024) Identification and mapping of QTLs for late blight resistance in the wild tomato (Solanum pimpinellifolium) accession PI 270442 via selective genotyping. Front Plant Sci 15. 10.3389/fpls.2024.1482241 10.3389/fpls.2024.1482241 PMC11604435 39619844

[CR63] Truco MJ, Ashrafi H, Kozik A, van Leeuwen H, Bowers J, Reyes Chin Wo S, Stoffel K, Xu H, Hill T, Van Deynze A, Michelmore RW (2013) An ultra high-density, transcript-based, genetic map of lettuce. G3 3:617–631 23550116 10.1534/g3.112.004929 PMC3618349

[CR64] USAblight.org (2019) Recent US genotypes. NC State. https://usablight.org/about-late-blight/recent-us-genotypes/. Accessed 17 May 2019

[CR65] Voorrips R (2002) MapChart: software for the graphical presentation of linkage maps and QTLs. J Hered 93:77–78. 10.1093/jhered/93.1.77 12011185 10.1093/jhered/93.1.77

[CR66] Wadl PA, Olukolu BA, Branham SE, Jarret RL, Yencho GC, Jackson DM (2018) Genetic diversity and population structure of the USDA sweetpotato (*Ipomoea batatas*) germplasm collections using GBSpoly. Front Plant Sci 9:1166. 10.3389/fpls.2018.01166 30186293 10.3389/fpls.2018.01166 PMC6111789

[CR67] Wang S, Basten CJ, Weir BS, Zeng Z-B (2006) Windows QTL Cartographer 2.5. Department of Statistics, North Carolina State University, Raleigh

[CR68] Zhang C, Liu L, Wang X, Vossen J, Li G, Li T, Zheng Z, Gao J, Guo Y, Visser RGF, Li J, Bai Y, Du Y (2014) The *Ph-3* gene from *Solanum pimpinellifolium* encodes CC-NBS-LRR protein conferring resistance to *Phytophthora infestans*. Theor Appl Genet 127(6):1353–1364. 10.1007/s00122-014-2303-1 24756242 10.1007/s00122-014-2303-1 PMC4035550

[CR69] Zhi X, Shu J, Zheng Z, Li T, Sun X, Bai J, Cui Y, Wang X, Huang Z, Guo Y, Du Y, Yang Y, Liu L, Li J (2021) Fine mapping of the *Ph-2* gene conferring resistance to late blight (*Phytophthora infestans*) in tomato. Plant Dis 105(4):851–858. 10.1094/PDIS-03-19-0679-RE 33021912 10.1094/PDIS-03-19-0679-RE

