# Supplementary material for: Identification of late blight resistance QTLs in an interspecific RIL population of tomato via genotyping-by-sequencing
Source: Mol Breed. 2025 Apr 8;45(4):43. doi: 10.1007/s11032-025-01560-6 (PMC11979090; doi:10.1007/s11032-025-01560-6)
Supplement: Supplementary file 1 — Supplementary file1 (DOCX 2133 KB) [file 11032_2025_1560_MOESM1_ESM.docx]

**LBRQTL-1.2**

**LBRQTL-1.1**

**LBRQTL-1.3**

**1.2**

**LBRQTL-10.1**

**10.2**

**10.1**

**LBRQTL-12**

**Supplementary Fig. 1** Genetic linkage bin map of tomato with 1,195 SNPs (markers shown are representative SNPs of each genetic recombinant bin). Genetic distance is shown on the left of each chromosome. QTL locations are shown with red bars on the left of chromosomes
