# Supplementary material for: Identification of late blight resistance QTLs in an interspecific RIL population of tomato via genotyping-by-sequencing
Source: Mol Breed. 2025 Apr 8;45(4):43. doi: 10.1007/s11032-025-01560-6 (PMC11979090; doi:10.1007/s11032-025-01560-6)
Supplement: Supplementary file 2 — Supplementary file2 (DOCX 17 KB) [file 11032_2025_1560_MOESM2_ESM.docx]

**Supplementary Table S1** Summary of genomic regions with significantly skewed segregation from Mendelian ratio shown by chi-square analysis (p < 0.01). Pr^NC^ = allele frequency of NC EBR-2 allele in the 122 RILs

| **Flanking markers** | **Chr #** | **Genetic location (cM)** | **Genetic distance (cM)** | **Physical distance (Mbp)** | **Number of**  **NC allele** | **Number of PI allele** | **Missing data** | **Pr^NC^** | ***X^2^* (1:1)** |
| --- | --- | --- | --- | --- | --- | --- | --- | --- | --- |
| SL3.0ch10_4207677 | 10 | 24.3 | 53.8 | 61.32 | 45 | 73 | 4 | 0.37 | 6.64 |
| SL3.0ch10_65525731 | 10 | 78.1 |  |  | 30 | 88 | 4 | 0.25 | 28.51 |
| SL3.0ch02_47284732 | 2 | 64.2 | 17.6 | 4.20 | 44 | 76 | 2 | 0.36 | 8.53 |
| SL3.0ch02_51482622 | 2 | 81.8 |  |  | 44 | 74 | 4 | 0.36 | 7.63 |
| SL3.0ch01_88454666 | 1 | 87.6 | 13 | 2.33 | 75 | 45 | 2 | 0.61 | 7.5 |
| SL3.0ch01_90779666 | 1 | 100.6 |  |  | 76 | 46 | 0 | 0.62 | 7.38 |
| SL3.0ch03_7588009 | 3 | 35.2 | 25.8 | 54.83 | 76 | 45 | 1 | 0.62 | 7.94 |
| SL3.0ch03_62415399 | 3 | 61 |  |  | 75 | 46 | 1 | 0.61 | 6.95 |
| SL3.0ch08_3614841 | 8 | 25.9 | 5.5 | 50.84 | 77 | 45 | 0 | 0.63 | 8.39 |
| SL3.0ch08_54451404 | 8 | 31.4 |  |  | 75 | 46 | 1 | 0.61 | 6.95 |
| SL3.0ch11_436099 | 11 | 2.1 | 53.3 | 6.83 | 74 | 45 | 3 | 0.61 | 7.07 |
| SL3.0ch11_7262409 | 11 | 55.4 |  |  | 75 | 45 | 2 | 0.61 | 7.5 |
| SL3.0ch11_53609442 | 11 | 78.4 | 1.7 | 0.52 | 75 | 45 | 2 | 0.61 | 7.5 |
| SL3.0ch11_54128668 | 11 | 80.1 |  |  | 75 | 44 | 3 | 0.61 | 8.08 |
