# Supplementary material for: Identification of late blight resistance QTLs in an interspecific RIL population of tomato via genotyping-by-sequencing
Source: Mol Breed. 2025 Apr 8;45(4):43. doi: 10.1007/s11032-025-01560-6 (PMC11979090; doi:10.1007/s11032-025-01560-6)
Supplement: Supplementary file 3 — Supplementary file3 (DOCX 20 KB) [file 11032_2025_1560_MOESM3_ESM.docx]

**Supplementary Table S2** Disease-related genes within the PI 270443 LB-resistance QTL regions identified in this study*. Gene locations and descriptions are based on SL3.0 and ITAG3.2 annotation, respectively

| QTL | Chr | Position(bp) | Gene ID | Description |
| --- | --- | --- | --- | --- |
| LBRQTL-10.1  (LBRQTL-10) | 10 | 63747603 | Solyc10g083920 | Serine/threonine protein phosphatase 2A |
|  | 10 | 63863103 | Solyc10g084060 | Cysteine-rich TM module stress tolerance protein |
|  | 10 | 63875845 | Solyc10g084085 | Disease resistance protein (TIR-NBS-LRR class) |
| LBRQTL-10.2  (LBRQTL-10) | 10 | 63996519 | Solyc10g084250 | Clade VIII lectin receptor kinase |
|  | 10 | 64075904 | Solyc10g084380 | WRKY transcription factor 44 |
|  | 10 | 64081078 | Solyc10g084400 | Glutathione S-transferase family protein |
|  | 10 | 64357086 | Solyc10g084840 | Pathogenesis-related thaumatin family protein |
|  | 10 | 64369581 | Solyc10g084860 | Clade XI lectin receptor kinase |
|  | 10 | 64435220 | Solyc10g084960 | Glutathione S-transferase-like protein |
|  | 10 | 64537390 | Solyc10g085110 | LRR receptor-like serine/threonine-protein kinase |
|  | 10 | 64539218 | Solyc10g085120 | Receptor-kinase |
|  | 10 | 64745279 | Solyc10g085440 | Serine/threonine protein kinase |
|  | 10 | 64745441 | Solyc10g085450 | Serine/threonine protein kinase |
|  | 10 | 64748798 | Solyc10g085460 | NBS-LRR resistance protein |
|  | 10 | 64918722 | Solyc10g085700 | Serine/threonine protein phosphatase 7 |
|  | 10 | 64986804 | Solyc10g085780 | NBS-LRR disease resistance protein |
|  | 10 | 65027135 | Solyc10g085850 | S-locus lectin protein kinase family protein |
|  | 10 | 65087310 | Solyc10g085960 | Pathogenesis-related protein 1 |
| LBRQTL-1.1 | 1 | 78861952 | Solyc01g079865 | Calcium/calmodulin-dependent Ser/Thr-protein kinase |
| LBRQTL-1.2 | 1 | 82330610 | Solyc01g087510 | LRR receptor-like kinase family protein |
|  | 1 | 82779327 | Solyc01g088060 | NBS-LRR resistance protein |
|  | 1 | 83308354 | Solyc01g088680 | Disease resistance protein (TIR-NBS-LRR class) |
|  | 1 | 83530181 | Solyc01g089960 | WRKY transcription factor 12 |
|  | 1 | 83996207 | Solyc01g090430 | NRC1 (NB-LRR family) |
| LBRQTL-1.3 | 1 | 86119625 | Solyc01g094835 | Serine/threonine-protein kinase |
|  | 1 | 86374134 | Solyc01g095100 | WRKY transcription factor 22 |
|  | 1 | 86507524 | Solyc01g095330 | LRR protein kinase family protein |
|  | 1 | 86649245 | Solyc01g095560 | Cysteine proteinases superfamily protein |
|  | 1 | 86714902 | Solyc01g095630 | WRKY transcription factor 41 |
|  | 1 | 88037596 | Solyc01g097240 | Pathogenesis-related protein PR-4 |
|  | 1 | 88055015 | Solyc01g097270 | Pathogen-induced protein |
|  | 1 | 88058744 | Solyc01g097280 | Pathogenesis-related protein PR-4 |
|  | 1 | 88774939 | Solyc01g098370 | LRR receptor-like kinase family protein |
|  | 1 | 88984140 | Solyc01g098680 | LRR receptor-like protein |
|  | 1 | 88989482 | Solyc01g098690 | LRR receptor-like protein |
| LBRQTL-12 | 12 | 64929580 | Solyc12g088670 | Cysteine protease CYP1 |
|  | 12 | 65113836 | Solyc12g088950 | LRR receptor-like protein kinase family |

*QTLs with the largest PVE are shown in bold
